# Workplace Health in Kentucky: A Statewide Comparison

**DOI:** 10.3390/ijerph18105473

**Published:** 2021-05-20

**Authors:** Gretchen Macy, Jacqueline Basham, Cecilia Watkins, Vijay Golla

**Affiliations:** 1 Department of Public Health, Western Kentucky University, Bowling Green, KY 42101, USA; Jacqueline.basham@wku.edu (J.B.); cecilia.watkins@wku.edu (C.W.); 2 Department of Life Sciences, Texas A&M University-San Antonio, San Antonio, TX 78224, USA; Vijay.golla@tamusa.edu

**Keywords:** keyword, workplace health promotion, Total Worker Health^®^, occupational safety and health

## Abstract

The objectives of this study were to assess the state of Kentucky’s workplace health promotion and occupational safety and health programs, to ensure the ability to comprehend any possible trends over the past six years in the state’s progress in offering workplace health promotion and health protection programs, to compare the results of this survey with the 2013 Kentucky state-wide assessment, and to identify gaps in Kentucky’s workplace health promotion and occupational safety and health based on Total Worker Health^®^ (TWH) concepts. Using Qualtrics research software, the Workplace Health in America assessment was sent to companies located in Kentucky and having 10 or more employees. Participants were identified using Dun and Bradstreet’s Hoover’s database. The results showed that, as with the 2013 survey, larger workplaces significantly were more likely to offer workplace health promotion programs than smaller companies (X^2^ = 24.30; *p* < 0.001). However, more companies (78%) reported offering programs compared to the 2013 assessment (49%). Given the results of the current study as compared to the statewide assessment conducted in 2013, Kentucky’s WHP is moving in a positive direction; yet, there is still much to be done. There remains a strong need to provide cost-effective and accessible resources for all elements of TWH to small workplaces.

## 1. Introduction

Chronic diseases, including heart disease, cancer, chronic lung disease, stroke, Alzheimer’s disease, diabetes, and chronic kidney disease continue to be the leading causes of death and disability in the United States, costing $3.5 trillion in annual health care costs [1]. The key lifestyle risks for these chronic diseases are tobacco use, poor nutrition, lack of physical activity, and excessive alcohol use [1]. According to America’s Health Rankings Annual Report [2], Kentucky ranks 50 out of 50 in physical inactivity, cancer deaths, and preventable hospitalizations. Kentucky also ranks 49 out of 50 in smoking and frequent mental health distress, while ranking 46 out of 50 in both drug deaths and obesity.

The labor force statistics [3] report that approximately 131 million Americans work full time. Due to the substantial amount of time individuals spend at work, the health of employees could be impacted by the environment of the workplace. Workplace policies, programs, and practices that support a culture of health have the potential to influence workers to make better health behavior choices [4].

The Centers for Disease Control and Prevention’s National Institute for Occupational Safety and Health’s (CDC/NIOSH) Total Worker Health^®^ (TWH) program is the strategic coordination of policies, programs, and practices that integrate health promotion and health protection to promote workers’ well-being in the workplace. The TWH concept addresses health issues that affect workers at both home and work [5].

Due to the growing research of the effectiveness of TWH [4] and the necessity to continue advancing the concept of TWH [5], the need to assess workplace health promotion programs and occupational safety and health programs has increased over the past few years. By assessing the concepts of TWH, as well as the quality of the concepts offered related to occupational safety and health in Kentucky, gaps may be identified and interventions developed and implemented to improve the overall health of Kentucky workers. In a case study conducted in rural Kentucky [6], results found that improved knowledge of participants about the need and importance of integration, as well as more comprehensive interventions that included management, were needed to promote the TWH concept. Additionally, there has been a call for future assessments to compare data sets in a systematic process [7,8,9].

This study, using the same instrument as the national 2017 Workplace Health in America (WHA) Survey [10], was conducted in the state of Kentucky with several goals in mind. The first goal included assessing the state of Kentucky’s workplace health promotion and occupational safety and health programs, which involved administering a state-wide survey. In order to review Kentucky’s efforts in providing health promotion and health protection at the workplace, another goal was to compare the results of this state-wide survey to the national 2017 WHA assessment [7,8]. To ensure the ability to comprehend any possible trends over the past six years in the state’s progress in offering workplace health promotion and health protection programs, a third goal was to compare the results of this survey with the 2013 Kentucky state-wide assessment. By tracking the progress and remaining gaps within the state, more targeted, quality interventions may be developed that aim to improve the health of Kentucky workers. The final goal of this study was to identify gaps in Kentucky’s workplace health promotion and occupational safety and health based on TWH concepts. According to Kentucky’s Vision for Worksite Wellness [11], “All public and private organizations will provide access to health food and physical activity opportunities to employees and their families through comprehensive worksite wellness programs”. At the time of the 2013 study, it was found that approximately 48% of employers in Kentucky offered some element of a workplace wellness program, with about 6% offering a comprehensive program [8]. The percentage of Kentucky workplaces offering elements of workplace health promotion (WHP) programs and comprehensive WHP programs were lower than the national percentages, with nearly half of all employers in the U.S. offering some element of WHP programs and 17% offering comprehensive WHP [7]. This research will assess the progress made over the last 6 years toward Kentucky’s vision for workplace wellness, as well as compare the percentages of workplaces offering elements of and comprehensive WHP to the national percentages.

## 2. Materials and Methods

Information on workplace health promotion program administration, occupational safety and health, disease management, incentives, work–life policies, barriers to implementation, evidence-based strategies, and health screenings was collected by utilizing the *Workplace Health in America* (WHA) survey [7]. This tool was selected due its inclusion of questions related to the quality and effectiveness of workplace health promotion (WHP) and occupational safety and health programs, in addition to emerging issues in workplace health, *Total Worker Health*^®^ concepts, and traditional elements of WHP. The survey includes 204 core items and 41 supplemental items and was designed by a steering committee of experts in workplace health promotion (WHP) led by the Centers for Disease Control and Prevention (CDC) [10].

The Dun and Bradstreet Hoover’s database was used to identify eligible participating workplaces. All businesses that contract with government agencies, as well as C corporations, partnerships, and LLCs, are required to have a data universal numbering system (DUNS) number. These unique identifiers, as well as company profiles (location, company size, industry, personnel), are included in the Hoover’s database. The database houses information for approximately 100,000 workplaces in Kentucky. Once a company was identified for inclusion in the study, individuals at that workplace with titles related to workplace wellness/health promotion, occupational safety and health, human resources, upper management, and president were identified as the points of contact to receive the survey participation request. Personnel with these titles were chosen based on the assumption that these individuals would be the most knowledgeable about WHP offerings. Prior to the dissemination of the electronic survey via the Qualtrics survey software, an email was sent to each eligible workplace inviting participation and alerting that a survey would soon follow. After the initial survey was sent, one follow-up email was sent per week for a total of 6 weeks. To be consistent with the national sample, the goal was a 10% participation rate. The sample included specific workplaces, rather than parent companies, to remain consistent with past national samples [7]. Inclusion criteria for workplaces included being located in the state of Kentucky and having 10 or more employees. Workplaces were further categorized based on size (10–24 employees, 25–49 employees, 50–99 employees, 100–249 employees, 250–499 employees, 500–749 employees, 750–999 employees, and 1000 employees or more). Additionally, workplaces were classified according to the North American Industry Classification System sectors.

This was a one-time, cross-sectional assessment. At the conclusion of the survey, participating workplaces had the option to enter a drawing to receive one training at no cost. Based on the findings of the survey, a priority area was identified and training was developed to address related gaps in occupational safety and health. Potential areas were the integration of health promotion and health protection, comprehensive workplace health promotion, various aspects of occupational safety and health, etc. Fifteen workplaces opted to be entered into the drawing to receive the training administered by the research team.

The WHA instrument was derived from a series of national workplace surveys. Key measures included the following: elements of a comprehensive WHP program, a workplace health promotion program administration, occupational safety and health, disease management, incentives, work–life policies, barriers to implementation, evidence-based strategies, and health screenings. Workplace demographics were also measured through a series of questions related to size based on number of employees, industry category, the percentage of the workforce that were full time employees (FTEs) and the percentage that were part time employees (PTEs), and whether the workplace was located at the company headquarters [7].

Statistical Package for the Social Sciences (SPSS) 26 and Qualtrics research software were used to analyze the data. Frequencies, graphs, and tables were used to describe the demographic characteristics. Descriptive statistics (means/percentages), standard errors, and 95% confidence intervals were calculated for each. Missing or incomplete data were removed from the analysis. A scale was created to analyze the comprehensiveness of WHP programs that included the 5 elements of a comprehensive WHP program: health education, links to employee assistance programs (EAPs), supportive physical and social environments, integration of health promotion into the organization’s culture, and employee screenings. Analysis of Variance (ANOVA) with post hoc least square difference (LSD) analysis was used to determine differences in means among the workplace by size and industrial classification codes.

## 3. Results

Three hundred and sixty-five out of 1400 randomly selected companies completed a portion of the assessment beyond the background information for a 13% overall response rate. Of the 168 workplaces providing information about employee demographics, 19% had less than 50 full time employees (FTEs), 16% had between 50–99 FTEs, 27% had between 100–249 FTEs, 17% of companies had between 250–499 FTEs, and 21% had over 500 FTEs. Seventy-four percent reported being located at the organization headquarters. The majority of the workplaces were for profit (See Table 1).

Of the workplaces providing information on industry type, classifications were manufacturing (31%), other services (16%), health care and social assistance (15%), educational services (13%), transportation (8%), professional and technical services (6%), retail/wholesale (3%), public administration (3%), mining (2%), agriculture (1%), arts/entertainment (1%), administrative support/waste management/remediation services (1%), and food services (1%) (See Table 1). The majority (96%) of respondents indicated that their companies provided health insurance, either full-time or part-time coverage, to FTEs. Most participants reported that FTEs were asked to pay “about the same” proportion of personal health insurance premiums compared to the last 12 months, while 28% reported having to pay more and 6% reported that their proportions were lower. Only 35% of workplaces reported offering personal health insurance to part time employees (PTEs) (See Table 1).

Approximately 78% of the participating companies reported having a workplace health promotion (WHP) program. Workplaces with a greater number of FTEs were more likely to have a WHP program than smaller companies. Of the participating companies with over 500 FTEs, 100% reported having a WHP program, whereas only 30% participating businesses with 10–24 FTEs reported having a WHP program. Additional information regarding established WHP programs by workplace size is shown in Table 1.

Thirty-four percent of companies reported having a WHP program for more than 10 years, while only 3% of companies reported having a WHP for less than one year (Table 1).

The largest group of WHP programs (54%) were managed by staff employees, while 26% were managed by a third-party vendor and 21% were managed by the health insurance provider. Larger organizations were more likely to report that an employee managed the WHP program than smaller companies, with 39% of organizations with greater than 500 FTEs reporting employee management of the WHP program as compared to none of the organizations with 10–24 FTEs. Most organizations (81%) reported that there was at least one person assigned the responsibility for the WHP program (Table 1). Again, larger organizations were more likely to report that there was at least one person assigned the responsibilities of the WHP program than smaller companies, with 29% of organizations with greater than 500 FTEs reporting at least one employee responsible for the WHP program as compared to only 1 of the organizations with 10–24 FTEs.

Sixty-six percent of organizations reported that a health risk assessment (HRA) was offered to employees in the last 12 months. Most organizations provided either results or provided both results and feedback and education for the identified risk factors (74%). Among participating organizations, 42% reported that the employer offered the HRA, while 28% were offered it through the health insurance plan, and 21% were offered it by a third-party vendor (Table 2).

The majority of organizations (88%) reported commitment and support to WHP from senior leadership. All reporting workplaces with less than 50 employees reported senior leadership commitment. Organizations also reported support from middle management, with 86% of participants indicating that middle management was visibly committed to employee health and safe work environments (Table 2). No differences were observed according to workplace size on the commitment of senior management or middle management. Larger companies were more likely to report a demonstrated organizational commitment to WHP than smaller companies, with 50% of companies with >500 FTEs indicating the commitment as compared to 56% of the companies with 250–499 FTEs, 31% of companies with 50–99 FTEs, and 20% of companies with 1–19 FTEs.

The majority of workplaces (86%) had annual goals and objectives for WHP. Larger organizations were more likely to report goals and objectives for WHP than smaller companies, with all organizations with 500 or more FTEs reporting goals and objectives for WHP compared to no organizations with 10–24 FTEs.

Approximately 79% of all workplaces offered incentives to employees to increase participation in WHP activities. Workplaces with a greater number of FTEs were more likely to offer incentives than workplaces with fewer FTEs. Almost 90% of organizations with more than 500 FTEs offered incentives for WHP participation compared to 33% of organizations with 25–49 FTEs. Almost 40% of workplaces felt that the incentives were effective or extremely effective in achieving the intended outcome (Table 2).

The majority of workplaces (72%) reported the use of data and ongoing evaluations to show WHP success. Again, larger workplaces were more likely to report ongoing evaluation than smaller workplaces.

The five key elements of a comprehensive WHP program are health education (e.g., skills development; behavior-change classes; and awareness building, such as brochures or posters), links to related employee services (e.g., referral to employee assistance programs (EAPs); supportive physical and social environments for health improvement (e.g., tobacco-free policies, subsidized gym membership); the integration of health promotion into the organization’s culture (e.g., health promotion as part of a business mission statement); and employee screenings with adequate treatment and follow-up (e.g., HRAs and biometric screenings) [7].

Almost 70% of the participating organizations reported offering health education programs. Larger companies were more likely to offer these elements than smaller companies. Companies with more than 500 FTEs were most likely to offer health education as part of the WHP program. Approximately two-thirds of participating organizations offered supportive social and physical environments. Half of all companies with fewer than 50 FTEs reported supportive physical and social environments for health improvement compared to 74% of companies with 100–249 employees and 96% of companies with more than 500 or more employees. The integration of health promotion into the organization’s culture was reported by 64% of respondents. Thirteen percent of companies with less than 25 employees, 42% of workplaces with 50–99 FTEs, and 82% of all companies with over 500 employees reported having this element as part of their WHP program. Most workplaces (84%) had linkages to related programs such as EAPs. All companies with more than 500 employees reported providing links to related employee services as compared to only 50% of companies with less than 25 employees. Companies with fewer than 25 employees were the least likely (30%) to report employee screenings with adequate treatment and follow-up as part of the WHP program, while companies with more than 500 employees were most likely (86%) to report the offering of this element. (See Table 2).

To further analyze the comprehensiveness of WHP programs, a scale was created that included the five elements of a comprehensive WHP program: health education, links to EAPs, supportive physical and social environments, the integration of health promotion into organization’s culture, and employee screenings. The scores ranged from 0–5, with higher scores indicating a greater number of elements of a comprehensive WHP program reported. The mean score for all workplaces was 3.48 (SD = 1.80). ANOVA analysis showed a statistically significant difference among the groups (F = 7.18; *p* > *0*.001), with larger workplaces offering more elements of comprehensive WHP than smaller companies.

This study had several limitations. The response rate of 13% could potentially affect external validity. There are possible differences between those workplaces that responded to the survey and those that did not. Some data may have been under- or over-reported due to the self-reporting nature of the instrument. The respondents of the survey may have also impacted the generalizability of the results.

## 4. Discussion

Trends such as an aging workforce, obesity, and increases in chronic health conditions, coupled with occupational injuries and accidents, are negatively impacting workplaces and impeding their ability to stay financially viable and maintain a healthy workforce [12]. Previous findings have shown that workplaces with established cultures of health have a healthier and safer workforce. Additionally, these companies tend to be more financially productive and maintain recruitment advantages [13,14,15].

This study aimed to determine if progress had been made in establishing cultures of health within Kentucky by comparing a survey from 2013 to the most recent survey administered. The distribution of participants in this study according to workplace size are similar to the 2013 Kentucky Statewide Assessment, with 19% of the participants having fewer than 50 FTEs [12], and the national WHA assessment, where 41% of all participating companies had fewer than 25 FTEs [7,8]. Results from the comparison show that Kentucky has seen improvements in the number of workplaces offering WHP programs. When comparing the findings of the current study to the previous assessment of WHP in Kentucky [12], 49% (*n* = 90) indicated that a WHP program was in place in the organization. Among those reporting an established WHP program, the majority (94%; *n* = 85) indicated that their organization had offered the WHP program for less than 5 years. The findings from the current study also showed that this sample of Kentucky workplaces were more likely to offer a health or wellness program than the nationally representative sample of 46% [7]. This is further supported by 94% of the workplaces with established WHP programs reporting their program as less than five years old. However, small businesses within Kentucky still seem to be struggling, with 40% of those employing less than 100 people indicating that they do not have a WHP program. These findings are consistent with both the findings from the 2013 Kentucky Statewide Assessment and the national WHA study [6,11]. This is especially important in Kentucky, since most of the labor force, 50.4%, is employed by establishments of less than 100 people [16].

Prior research has shown that a key aspect of encouraging safety and health in the workplace is the commitment shown by senior leadership members [17]. More specific studies have found that safety and health behaviors were positively impacted by the commitment to safety shown by management, supervisors, and co-workers [18]. The most recent survey results indicated that organizations with a WHP program reported commitment and support from senior leadership (66%), as well as from middle management (87%). Of the participating workplaces in the 2013 study, 34% (*n* = 53) indicated a demonstrated organizational commitment and support of WHP at all levels of management. Again, these findings are consistent with the national sample, which indicated that senior leadership (84%) and middle management (83%) were committed to employee safety and health [7]. The commitment indicated within the current study shows that workplaces choosing to adopt WHP programs within Kentucky are doing so in a way that is likely to encourage both the safety and health of their workers.

In addition to leadership commitment, other key principles of a WHP program include incentives that showcase the program as a positive benefit of the company and evaluation to determine if the program is accomplishing its purpose [19]. In the 2013 study, approximately 43% of workplaces reported the use of incentives to increase employee participation. Nine out of ten, or 90%, of the participating workplaces with >500 FTEs reported the use of incentives as compared to 56% of the companies with 250–499 FTEs (*n* = 16), 30% of companies with 20–49 FTEs (*n* = 43), and 20% of workplaces with 1–19 FTEs (*n* = 15) [12]. Results from the most recent survey, as well as the 2013 survey, showed workplaces with more FTEs were significantly more likely to offer incentives than workplaces with fewer FTEs. The WHA study also showed that larger workplaces were more likely to offer incentives than smaller workplaces [7].

Important to note is that not all companies with WHP programs reported setting goals and objectives, which makes it hard for evaluation to be completed. Larger companies were more likely to have set goals and objectives than smaller companies, which helps to explain this trend in evaluation. This same trend was also seen in the evaluation of the WHP programs to determine if the goals and objectives of the program were successful. In the 2013 study, 22% of workplaces (*n* = 34) reported ongoing evaluations of health promotion programming using multiple data sources. There were significant differences based on workplace size, with over two-thirds of large workplaces conducting evaluation with multiple data sources as compared to none of the smallest workplaces [12].

## 5. Conclusions

Given the results of the current study as compared to the statewide assessment conducted in 2013, WHP in Kentucky is moving in a positive direction; yet, there is still much to be done. There remains a strong need to provide cost-effective and accessible resources to small companies. These companies continue to make up the largest proportion of the workplaces in Kentucky and offer the fewest elements of workplace health promotion. In the future, additional resources and efforts need to be targeted at establishing collaborations between small businesses and outside organizations to provide affordable and user-friendly resources that improve the occupational safety and health of Kentucky’s workforce. The results of this study may also be used to inform additional research in other locations, especially locations with high percentages of small workplaces. Additional research is needed to fully understand the best way to meet the needs of workers, both nationally and internationally.

## Figures and Tables

**Table 1:** Workplace characteristics.

Element	Total # of Survey Responses	*n* (%)	
Demographics			
Number of FTEs	168		
10–24 FTEs		12 (7.1)	
25–49 FTEs		20 (11.9)	
50–99 FTEs		27 (16.1)	
100–249 FTEs		45 (26.8)	
250–499 FTEs		29 (17.3)	
≥500 FTEs		35 (20.8)	
Located at organization headquarters	192	143 (74.4)	
Business type	192		
For-profit		106 (55.2)	
Non-profit/governmental		49 (25.5)	
Non-profit/other		37 (19.3)	
Industry type	168		
Manufacturing		52 (30.9)	
Other services (except public administration)		26 (15.4)	
Health care and social assistance		25 (14.9)	
Educational services		21 (12.5)	
Transportation, warehousing, and utilities		14 (8.3)	
Professional, scientific, and technical services		10 (6.0)	
Retail/wholesale trade		5 (3.0)	
Public administration		5 (3.0)	
Mining, quarrying, and oil and gas extraction		3 (1.8)	
Agriculture, forestry, fishing, and hunting		2 (1.2)	
Arts, entertainment, and recreation		2 (1.2)	
Administrative support, waste management, and remediation services		2 (1.2)	
Accommodation and food service		1 (.6)	
Offered insurance to FTEs	216	207 (95.8)	
Full coverage		104 (48.1)	
Partial coverage		103 (47.7)	
No coverage		9 (4.2)	
Offered insurance to PTEs	167	59 (35.3)	
Change in employee proportion of insurance premiums compared to last year	185		
Premiums increased		51 (27.6)	
Premiums stayed about the same		123 (66.5)	
Premiums decreased		11 (5.9)	
Workplace health promotion (WHP)			
Established WHP program by workplace size	138	Yes	No
10–24 FTEs		3 (30.0)	9 (70.0)
25–49 FTEs		9 (64.3)	5 (35.7)
50–99 FTEs		15 (71.4)	6 (28.6)
100–249 FTEs		32 (82.1)	7 (17.9)
250–499 FTEs		20 (80.0)	5 (20.0)
≥500 FTEs		29 (20.8)	0 (0.0)
Total		108 (78.3)	30 (21.7)
Age of established WHP program	109		
Less than 1 year		3 (2.8)	
1 to 2 years		9 (8.3)	
3 to 5 years		37 (33.9)	
6 to 9 years		23 (21.1)	
10 or more years		37 (33.9)	
Management of WHP programs	117		
Staff employees		63 (53.9)	
Third party vendor		30 (25.6)	
Health insurance provider		24 (20.5)	
At least one person assigned to WHP	128	103 (80.5)	

**Table 2:** Worksites offering elements of organizational support and comprehensive WHP programs.

Element	Total # of Survey Responses	*n* (%)
Organizational support offered		
Employee HRA	165	109 (66.4)
HRA offered by:	103	
Employer		43 (41.8)
Health insurance plan		29 (28.1)
Third-party vendor		22 (21.4)
Did not know		9 (8.7)
Senior leadership demonstrated commitment and support to WHP	120	105 (87.5)
Middle management demonstrated commitment and support to WHP	117	100 (85.5)
Annual specific goals and objectives for WHP	63	54 (85.7)
Utilized incentives to increase participation	85	67 (78.8)
Effectiveness of incentive offered:	58	
Extremely effective		5 (8.6)
Effective		18 (31.0)
Somewhat effective		22 (37.9)
Not at all effective		2 (3.5)
Did not know		11 (19.0)
Ongoing evaluations and data to evaluate success	89	64 (71.9)
Elements of comprehensive WHP		
Health education programs	176	121 (68.8)
Supportive social and physical environment	183	134 (73.2)
Integration of WHP into the organization’s structure	163	105 (64.4)
Linkage to related programs like EAPs	173	145 (83.8)
Worksite health screening	178	118 (66.3)

## Data Availability

Data is available upon request.
